# Supplementary material for: A Plausible Link of TMPRSS2/ACE2/AR Signaling to Male Mortality during the COVID-19 Pandemic in the United States
Source: Pathogens. 2021 Oct 26;10(11):1378. doi: 10.3390/pathogens10111378 (PMC8618612; doi:10.3390/pathogens10111378)
Supplement: Supplementary file 1 [file pathogens-10-01378-s001.zip › Figure S1.pdf]

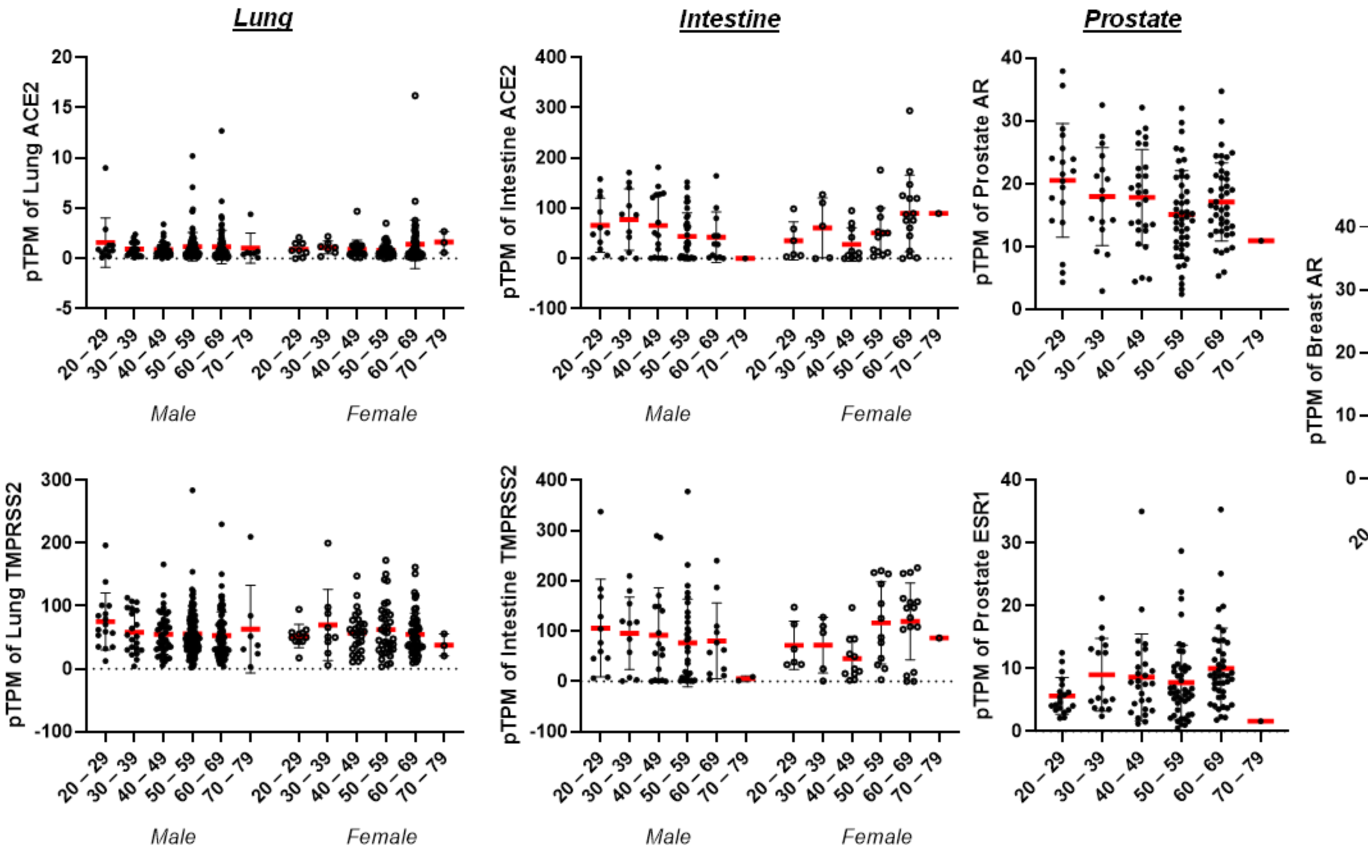

Figure S1. In vivo AR/TMPRSS2/ACE2 gene expression measured by RNA-seq analysis. Gene expression of TMPRSS2 and ACE2 in lung (**A**, *n* = 427), intestine (**B**, *n* = 137), prostate AR/ESR1 (**C**, *n* =152), and breast AR (**D,** *n* = 290) by age was analyzed with the tissue RNA-seq data retrieved from The Genotype-Tissue Expression (GTEx) project. The data were plotted as the mean value of the protein transcripts per million reads (pTPM).
